# Increased Expression of Maturation Promoting Factor Components Speeds Up Meiosis in Oocytes from Aged Females

**DOI:** 10.3390/ijms19092841

**Published:** 2018-09-19

**Authors:** Marketa Koncicka, Anna Tetkova, Denisa Jansova, Edgar Del Llano, Lenka Gahurova, Jana Kracmarova, Sarka Prokesova, Tomas Masek, Martin Pospisek, Alexander W. Bruce, Michal Kubelka, Andrej Susor

**Affiliations:** 1 Laboratory of Biochemistry and Molecular Biology of Germ Cells, Institute of Animal Physiology and Genetics, CAS, Rumburska 89, 277 21 Libechov, Czech Republic; Koncicka@iapg.cas.cz (M.K.); tetkova@iapg.cas.cz (A.T.); jansova@iapg.cas.cz (D.J.); Llano@iapg.cas.cz (E.D.L.); lveselovska@prf.jcu.cz (L.G.); kracmarova@iapg.cas.cz (J.K.); prokesovas@af.czu.cz (S.P.); kubelka@iapg.cas.cz (M.K.); 2 Department of Cell Biology, Faculty of Science, Charles University in Prague, Albertov 6, 128 43 Prague, Czech Republic; 3 Department of Genetics and Microbiology, Faculty of Science, Charles University in Prague, Albertov 6, 128 43 Prague, Czech Republic; masek@natur.cuni.cz (T.M.); martin.pospisek@natur.cuni.cz (M.P.); 4 Laboratory of early mammalian development, Department of Molecular Biology and Genetics, Faculty of Science, University of South Bohemia, Branisovska 1760, 370 05 Ceske Budejovice, Czech Republic; awbruce@prf.jcu.cz

**Keywords:** aging, oocyte, MPF, meiosis, translation, lamin A/C

## Abstract

The rate of chromosome segregation errors that emerge during meiosis I in the mammalian female germ line are known to increase with maternal age; however, little is known about the underlying molecular mechanism. The objective of this study was to analyze meiotic progression of mouse oocytes in relation to maternal age. Using the mouse as a model system, we analyzed the timing of nuclear envelope breakdown and the morphology of the nuclear lamina of oocytes obtained from young (2 months old) and aged females (12 months old). Oocytes obtained from older females display a significantly faster progression through meiosis I compared to the ones obtained from younger females. Furthermore, in oocytes from aged females, lamin A/C structures exhibit rapid phosphorylation and dissociation. Additionally, we also found an increased abundance of MPF components and increased translation of factors controlling translational activity in the oocytes of aged females. In conclusion, the elevated MPF activity observed in aged female oocytes affects precocious meiotic processes that can multifactorially contribute to chromosomal errors in meiosis I.

## 1. Introduction

The development of female germ cells (oocytes) is essential for sexual reproduction. Oocytes, arrested in meiotic prophase, undergo a major growth phase during their development in ovarian follicles. During this phase, they actively transcribe their genome; however, most derived mRNAs are stored in ribonucleoprotein particles to be used much later during the final stages of meiosis and early embryonic development. A unique property of the oocyte is that the final stages of meiosis (after prophase I) occur in the absence of de novo transcription. Consequently, regulation of mRNA stability and translation serves as the main driving forces behind oogenesis and early embryogenesis [1]. 

Mammalian oocytes undergo two successive cell divisions without an intermediate replicative phase. This brief period is called “meiotic maturation” and is crucial for the formation of an egg capable of being fertilized and for the generation of viable and euploid offspring. At the onset of meiosis I, the nuclear lamina is phosphorylated (namely lamin A/C; LMN A/C) and disassembled, leading to nuclear envelope break down (NEBD), chromosome condensation, and progressive reorganization of microtubules into a bipolar spindle [2]. At the end of meiosis I, the first asymmetric division occurs. 

Human and mouse oocytes are vulnerable to aging as the incidence of chromosome segregation errors (aneuploidy) reaches high levels in females/mothers of advanced age [3,4,5]. For example, in 20-year-old women, aneuploidy occurs in ~2% of matured oocytes; however, after 35 years of age aneuploidy increases to 35% [6,7]. Similarly, oocytes from aged mice display a significant increase in the incidence of aneuploidy. In three-month-old mice, aneuploidy occurs in 5% of cases; however, by 12 months of age this figure increases to 30–50% [4,8,9]. The majority of chromosome segregation errors are known to arise during the first meiotic cytokinesis [6,10]; however, the reasons why female meiosis shows this peculiar vulnerability to aging remains unclear. 

In this study, we present evidence for the aberrant timing of meiosis I in the oocytes derived from female mice of advanced age. Such age-associated abnormalities present as aberrations in nuclear envelope morphology as well as the precocious timing of NEBD and the formation of kinetochore-microtubule (K-MT) attachments, resulting in accelerated first polar body extrusion (PBE). Furthermore, we reveal that it is the overexpression of metaphase promoting factor (MPF) components associated with impaired translational machinery that leads to this phenotype.

## 2. Results

### 2.1. Meiosis I Is Accelerated in Oocytes from Females of Advanced Age

**Figure 1:**
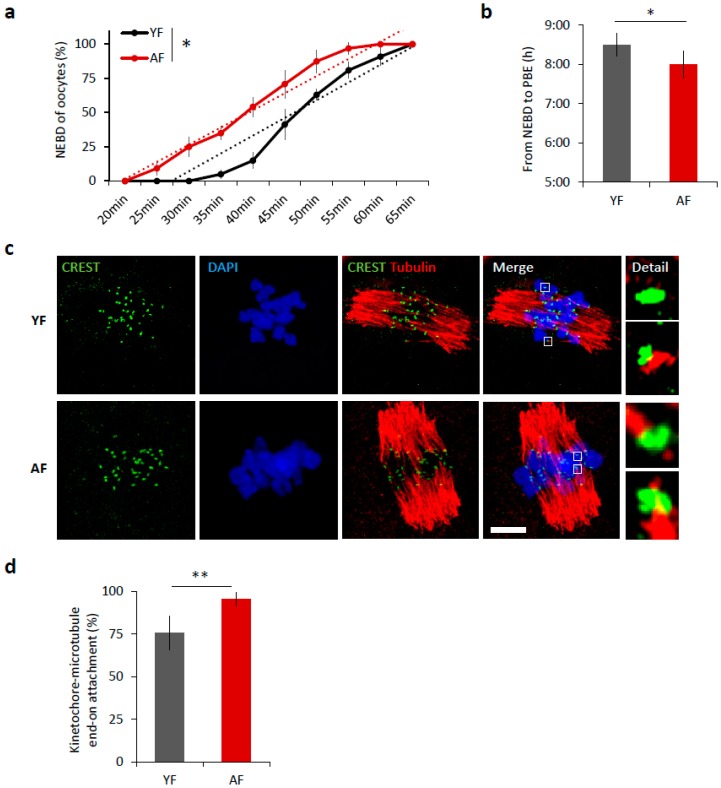
Meiosis I is accelerated in oocytes from females of advanced age. (**a**) Timing of nuclear envelope breakdown (NEBD) of oocytes isolated from young females (YF, *n* = 80; black line) and aged females (AF, *n* = 68; red line). Trend line is depicted by dot line. Data represent mean ± SD, *n* = 6, * *p* < 0.05, Student’s *t*-test. (**b**) Time from NEBD to polar body extrusion (PBE) in oocytes from YF (*n* = 80; t = 8:30 h) and AF (*n* = 68; t = 8 h). Data represent mean ± SD and data are from at least three experiments of biologically different samples. * *p* < 0.05, Student’s *t*-test. (**c**) Representative Z-projections from the assessment of cold stable attachments of kinetochore (KT) to microtubule (MT) imaged by confocal microscopy using CREST (green) and Tubulin (red) antibodies. Representative images from three experiments of biologically different samples are presented (scale bar, 10 µm). (**d**) The percentage of cold stable end-on MT to KT attachments in each age group averaged over multiple cells (*n* ≥ 15) 6 h post-IBMX-wash. Kinetochore-MT end-on attachments were quantified. The morphology of kinetochores analyzed is specified in detail. Data represent mean ± SD. ** *p* < 0.01, Student’s *t*-test.

It is well known that increased maternal age negatively affects oocyte quality [3,5]. We isolated oocytes from antral follicles and obtained an average of 22 fully grown GV oocytes per young mouse (YF; 2 months old) compared to 3 oocytes per aged female (AF; 12 months old). Following removal of IBMX from the culture medium, to restart meiosis I, 98.75% of selected oocytes from young females and 98.53% oocytes from aged females resumed meiosis (NEBD; Student’s *t*-test *p* = 0.9985). Of the cells that resumed meiosis, 84% of the young oocytes extruded polar body and reached MII in the 12 h period compared to 94% of AF oocytes (Student’s *t*-test *p* = 0.010809). Measurement of oocyte diameter did not show any differences between age groups (71.73 ± 1.5 and 72.31 *±* 1.6 µm, respectively, Student’s *t*-test *p* = 0.99743). To analyze the effect of maternal age on the progress of meiosis I, we compared the maturation of mouse oocytes from young females (YF; 2 months old) and aged females (AF; 12 months old). Time-lapse microscopy revealed that the oocytes from AF progress through meiosis I significantly 30 min faster than oocytes from YF (*p* < 0.05; Figure 1a,b). The oocytes in the AF group initiate nuclear envelope breakdown (NEBD) earlier (Figure 1a) and consequently polar body extrusion (PBE) also occurs earlier than in the YF group (*p* < 0.05; Figure 1b); manifest as a shortening of time between NEBD and PBE (Figure 1b). Next, we scored the attachment of individual cold-stable microtubules (MT) with end-on kinetochores in both age groups. We found that, during metaphase I, 6 h after releasing oocytes from prophase I (6 h post-IBMX-wash), the AF group had a higher number of stably attached kinetochores (95.5%) than the YF group (75.8%, *p* < 0.01) (Figure 1c,d). The larger number of stably end-on attached kinetochores in the AF group demonstrates that the progression through meiosis I was accelerated in the oocytes from the AF group. 

### 2.2. Dissociation of Nuclear Envelope Is Accelerated in the Oocytes from Aged Females 

**Figure 2:**
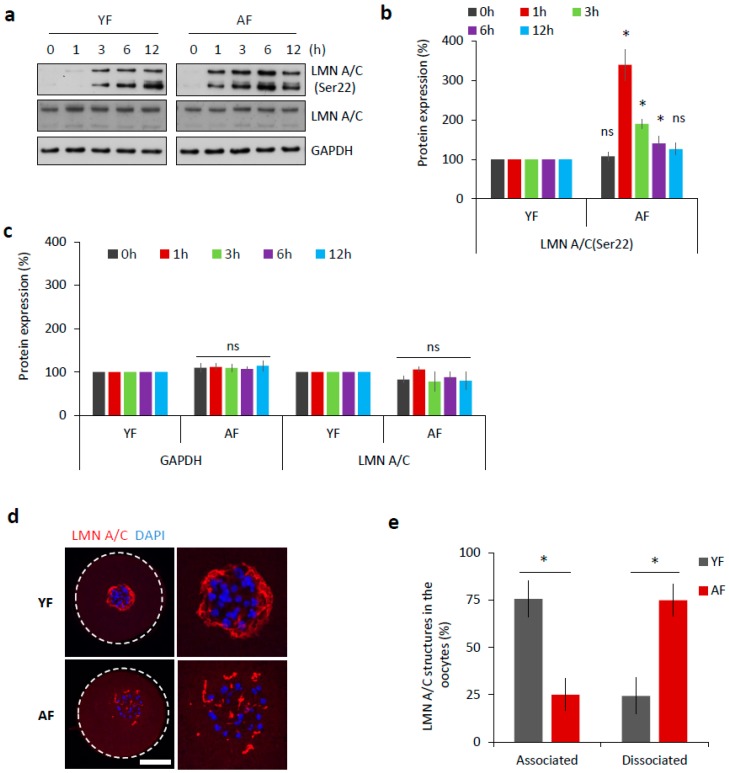
Dissociation of nuclear lamina is accelerated in the oocytes from the AF group. (**a**) Western blot analysis of phosphorylation status of LMN A/C (Ser22) at different time points during meiotic progression (0 h, GV; 1 h, post-NEBD; 3 h, post-NEBD; 6 h, post-NEBD/metaphase I; 12 h, post/NEBD/metaphase II). Antibodies against LMN A/C and GAPDH were used as loading controls. Representative images are from three experiments of biologically different samples. (**b**) Quantification of LMN A/C phosphorylation (Ser22) at different time points during meiotic maturation. Data are from three experiments of biologically different samples. Values obtained for the YF group were set as 100%. AF values from each antibody was compared between groups and same oocyte stage. Data represent mean ± SD. * *p* < 0.05, bars with ns are non-significant, Student’s *t*-test. (**c**) Quantification of GAPDH and LMN A/C protein expression at different time points during meiotic maturation. Data are from three experiments of biologically different samples. Values obtained for the YF group were set as 100%. AF values from each antibody was compared between groups and same oocyte stage. Data represent mean ± SD. * *p* < 0.05, bars with ns are non-significant, Student’s *t*-test. (**d**) Representative images of LMN A/C structures 3 h post-IBMX-wash (post-NEBD, scale bar 20 µm). The cortex of the oocyte indicated by the white dashed line. See Figure S1 for the LMN A/C localization and phosphorylation during oocyte meiotic progression and Figure S2 for electron microscopy images of the nuclear lamina. (**e**) Quantification of LMN A/C structures in the oocytes from different age groups post-NEBD (*n* ≥ 33 and three independent biological replicates). Data represent mean ± SD. * *p* < 0.05, Student’s *t*-test.

The abundant components of the nuclear envelope are lamin A and C (LMN A/C) [11]. Phosphorylation of these lamins at Serine-22 (Ser22) triggers the disassembly of the nuclear lamina, which is a prerequisite for nuclear envelope breakdown [12]. Therefore, we analyzed the phosphorylation of LMN A/C (Ser22) as a marker of meiotic progression. Oocytes from both age groups were analyzed by Western blotting at various time points relative to their initial isolation (i.e., after 0, 1, 3, 6, and 12 h). We found that the AF group had a significantly increased level of phosphorylated LMN A/C 1 h post-IBMX-wash (*p* < 0.05; Figure 2a,b). On the contrary, the YF group only had an abundant level of phosphorylated LMN A/C 3 h post-IBMX-wash (Figure 2a,b). Despite the observed different timing of LMN A/C phosphorylation between these two groups, the total/eventual level of LMN A/C remained constant (Figure 2a,c). 

It has been previously documented [13,14] that nuclear lamina structures can be still present at least a few hours after NEBD in mouse oocytes. By immunocytochemistry (ICC), we visualized LMN A/C structures during oocyte meiotic maturation (Figure S1a,b). Using specific antibodies, both total LMN A/C as well as phosphorylated LMN A/C (Ser22) were detected within the disrupted nuclear lamina structures in NEBD stage oocytes, 3 h post-IBMX-wash (Figure S1a,b). The observed lamina structures surrounded the chromosomal area, where the new spindle was due to be assembled (Figure S1c), but disappeared as meiosis progressed (Figure S1). When we compared 3 h post-IBMX-wash oocytes from both age groups, we found that the dissociation of the described LMN A/C structures was completed significantly faster in the AF group, at a time-point at which they still persisted in the YF oocytes (*p* < 0.05; Figure 2d,e).

Additionally, we imaged GV stage oocytes (oocytes with intact nucleus designated as germinal vesicle, GV) from both age groups by transmission electron microscopy and we were able to distinguish visible differences in the structure of the nuclear envelope in both groups. The nuclear membrane of AF oocytes presented an unique characteristic series of invaginations and decreased compactness (Figure S2a,b). The distinct morphology of the nuclear envelope in the AF oocyte group resulted in a significant increase in the circumference of the nuclear envelope (*p* < 0.01; Figure S2b). Moreover, the observed ultrastructural morphology of the nuclear lamina in AF oocytes resembled that reported in the nuclear phenotypes of other aged cells [15,16].

To conclude, in addition to the above-mentioned precocious timing in meiosis, observed in AF oocytes, we also observed a comparatively earlier phosphorylation of LMN A/C that was associated with a faster disassembly of nuclear lamina, thus affecting the timing of nuclear membrane breakdown, when compared to oocytes from the YF group.

### 2.3. CDK1 Activity Is Responsible for NEBD in Mouse Oocytes 

**Figure 3:**
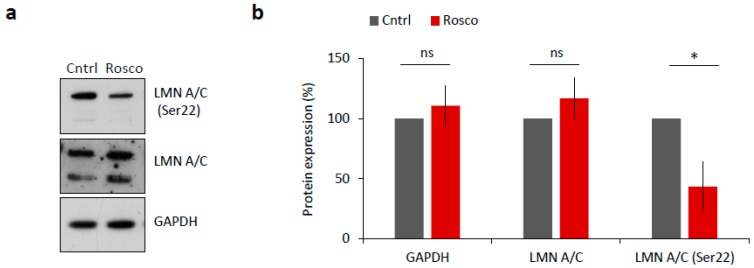
CDK1 is responsible for LMN A/C phosphorylation in mouse oocyte. (**a**) Western blot analysis of oocyte samples treated for 2 h after NEBD with 20 µM CDK1 inhibitor, Roscovitine (Rosco). Phosphorylation status of LMN A/C (Ser22) was detected using a specific antibody. Antibodies against LMN A/C and GAPDH were used as a loading control. (**b**) Quantification of total and phosphorylated LMN A/C after Roscovitine (Rosco) treatment. Protein levels were normalized in a way that non-treated controls are 100%. Data was derived from three experiments containing biologically different samples. Columns represent mean, ± SD; ns non-significant; * *p* < 0.05, Student’s *t*-test.

NEBD is reported to be driven by CDK1 (MPF) activity via phosphorylation of lamin proteins and subsequent lamina disassembly at the onset of meiotic resumption or mitosis [17,18]. To test whether LMN A/C were phosphorylated in a CDK1-dependent manner, we treated mouse oocytes with 20 µM Roscovitine (Rosco), a potent inhibitor of CDK1 activity, for 2 h after NEBD. We found significantly decreased levels of LMN A/C (Ser22) phosphorylation in oocytes treated with Rosco (*p* < 0.05; Figure 3a,b) versus controls, a result that is consistent with findings of [17].

Thus, our data confirm the functional involvement of the activated MPF in nuclear lamina disassembly through regulation of LMN A/C phosphorylation status. 

### 2.4. CDK1 Activity Is Increased in Mouse Oocytes from Aged Females

**Figure 4:**
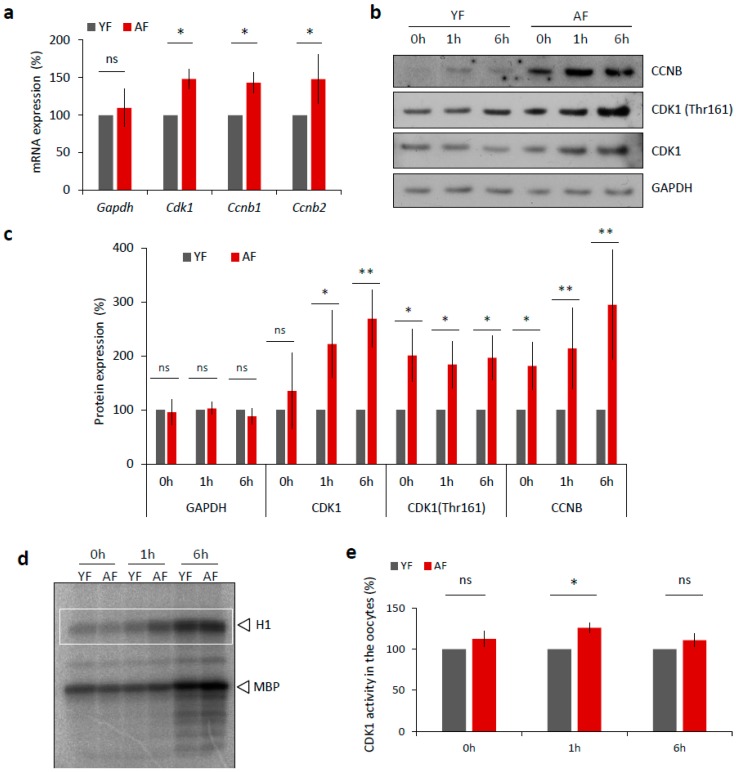
Expression of MPF components and its activity is increased in the oocytes from aged females. (**a**) RT-PCR quantification of mRNA coding for CDK1 and B-type cyclins, as well as loading control *Gapdh* in the GV oocytes (0 h) from different age groups. For quantification of total RNA content in oocytes from YF and AF groups see Figure S3a. Values obtained for the YF group were set as 100%. Data was derived from at least four experiments of biologically different samples. Columns represent mean; error bars ± SD; ns non-significant; * *p* < 0.05, Student’s *t*-test. (**b**) Western blot analysis of CDK1, CDK1 (Thr161) and CCNB during oocyte maturation (0 h, 1 h and 6 h) in the both age groups. See Figure S3b,c for the assessment of global translation in oocytes from YF and AF groups. (**c**) Quantification of MPF components, CDK1, its phosphorylation (Thr161), CCNB and GAPDH as a loading control. Values obtained for the YF group were set as 100%. From at least three experiments of biologically different samples. Columns represent mean ± SD; * *p* < 0.05; ** *p* < 0.01; bars with ns are non-significant; Student’s *t*-test. (**d**) Representative image of analysis of CDK1 activity (H1) in the oocytes after isolation (0 h), NEBD (1 h) and at metaphase I (6 h). Kinase assay was done with oocytes of both female age groups. CDK1 activity was measured towards histone H1 as external substrate, marked by white rectangle. (**e**) Quantification of CDK1 (H1 substrate) activity during oocyte maturation from YF and AF groups. Measurements originated from four experiments of biologically different samples. Values obtained for the YF group were set as 100%. Columns represent mean; error bars ± SD; ns non-significant; * *p* < 0.05; Student’s *t*-test.

We next examined, whether the expression of MPF components, that directly affect meiotic progression [19], differs between the YF and AF groups of mouse oocytes. Firstly, we isolated total RNA from transcriptionally silent GV staged oocytes from each group and performed quantitative RT-PCR mRNA expression analysis of the MPF component genes *Cdk1* and the B-type *Cyclins*. We found significantly increased levels of both *Cdk1* and *Cyclin B* transcripts in the oocytes from the AF group (Figure 4a) that were not reflected in the total RNA content (Figure S3a) nor in the expression level of *Gapdh* mRNA (Figure 4a). Next we analyzed the expression of MPF components at the protein level via Western blotting, and again we discovered a significant increase in the expression levels of CCNB and CDK1 proteins, specifically in the AF group of oocytes (Figure 4b,c). In addition to the use of a pan-CDK1 antibody, we also probed the oocyte samples with an antibody that specifically recognized phosphorylated (Thr161) CDK1, the enzymatically active form of the protein that is required for a functional MPF activity [19,20,21,22]. Again, we found increased phosphorylation status of CDK1 in the AF group versus the YF group of oocytes (Figure 4d,e). Consistently, an analysis of MPF activity, using a standard kinase assay, also revealed a significant increase in CDK1 activity at 1 h in the AF oocytes compared with the YF oocytes, at the time of NEBD (Figure 4d,e).

Taking these results together, we conclude that the activation of the MPF is significantly accelerated in the oocytes from aged females.

### 2.5. Translation of Positive Regulators of Translation Is Increased in the Oocytes from Aged Females

**Figure 5:**
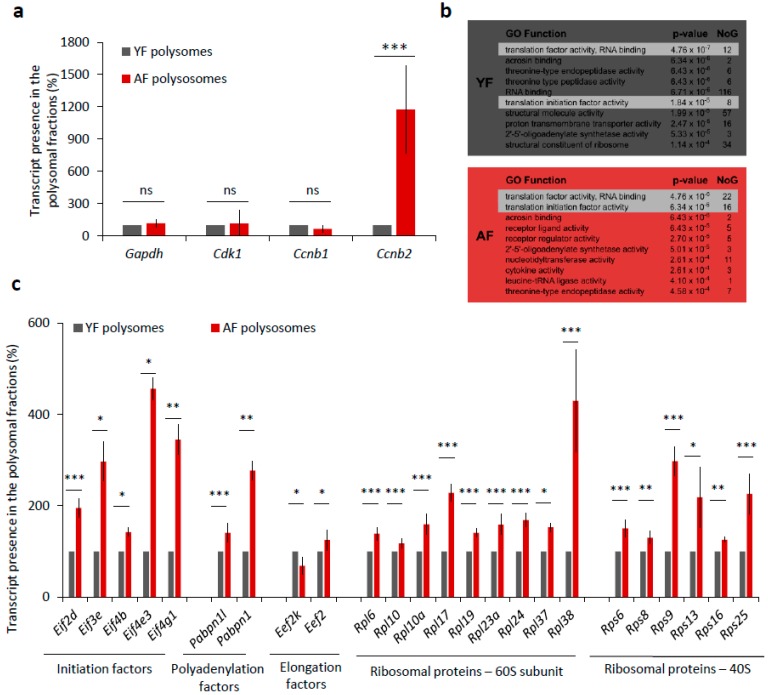
Translation of a number of translational factor components is increased in the oocytes from aged females. (**a**) mRNA abundance of MPF components, *Cdk1* and *Cyclins B* as well as *Gapdh* used as the control in the polyribosomal fractions. Percentage of reads from RNA-Seq. See Figure S3a for quantification of total RNA in oocytes from YF and AF groups. Three independent experimental datasets from biologically different samples. Values obtained for the YF group were set as 100%. Data columns represent mean; error bars, ± SEM; ns non-significant; *** *p* < 0.001; Student’s *t*-test. (**b**) Top 10 most enriched GO Function categories in polysome-bound mRNAs in YF (grey) and AF (red) oocytes determined by Gorilla. NoG (Number of Genes) denotes the number of genes in enriched categories. Highlighted lines represent translational functional categories. (**c**) Translational regulation of mRNA coding for different translational factors from polysomal fractions of YF and AF oocytes. Values obtained for the YF group were set as 100%. Data represent mean ± SEM; * *p* < 0.05; ** *p* < 0.01; *** *p* < 0.001; Student’s *t*-test.

De novo transcription in fully grown oocytes ceased, so we wondered whether the elevated levels of CCNB and CDK1 proteins in the oocytes from aged females were only due to higher transcript abundance or could also be related to higher translational activity. To experimentally address this question, we compared the incorporation of ^35^S-Methionine into nascently translated proteins in both the YF and AF groups of oocytes during maturation (Figure S3b), but we found no significant difference in the levels of global translation (Figure S3b,c). However, we also derived an RNA-seq dataset of mRNA polyribosomal occupancy that allowed us to detect and identify actively translated mRNAs in the two studied age groups of oocytes. Whilst the polysome occupancy was unchanged for mRNAs encoding GAPDH, CDK1, and CCNB1, we did intriguingly identify *Ccnb2*-derived transcripts enriched over 11-fold in polysomal fractions from AF compared to YF oocytes (Figure 5a). A further GO-term (gene ontology) enrichment analysis of polysome-bound mRNAs indicated a significant enrichment of mRNA coding for protein factors belonging to GO functional categories associated with translation initiation and regulation, specifically in the AF oocyte group (*p*-value = 4.76−6.34 × 10^−6^ for 38 individual mRNAs) (Figure 5b). Generally, this enrichment was higher and the respective categories contained more mRNAs in the AF group over the YF oocytes. We therefore systematically examined the polysome-bound mRNAs whose products are involved in the regulation of translation and revealed increased levels of mRNA coding for positive translation regulators, namely, eukaryotic translation initiation factors (eIF2D, eIF3E, eIF4B, eIF4E3, and eIF4G1), polyadenylation factors (PABPN1L and PABPN1), elongation factor (eEF2), and the number of ribosomal proteins (60S-RPL6, RPL10, RPL10A, RPL17, RPL19, RPL23A, RPL24, RPL37, RPL38; 40S-RPS6, RPS8, RPS9, RPS13, RPS16, and RPS25) in the AF group of oocytes (Figure 5c). Contrarily, we detected a decreased level of mRNA-polysome association for the elongation factor kinase (eEF2K), which is known to act as a suppressor of translational elongation (Figure 5c).

Overall, these results suggest that the translation of individual MPF components and of specific translational factors is elevated in AF oocytes, which is likely to result in changes in the physiology of oocytes from aged female mice. 

### 2.6. Elevated CDK1 Activity Is Responsible for Faster Meiosis I in Mouse Oocytes 

**Figure 6:**
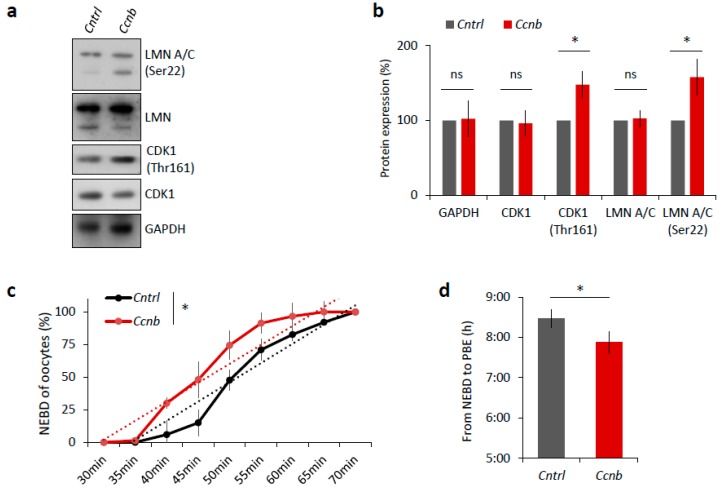

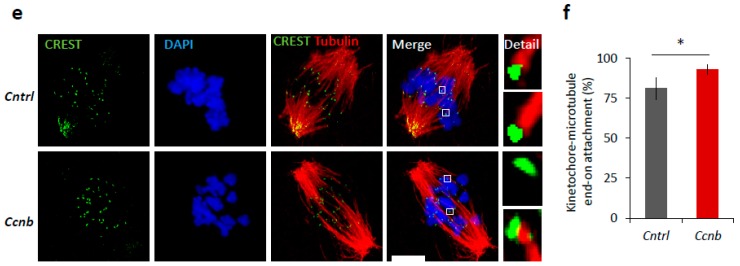
Elevated MPF activity is responsible for meiosis I acceleration in oocytes. (**a**) Western blot analysis of LMN A/C (Ser22), LMN A/C, CDK1 (Thr161), CDK1, and GAPDH in the post-NEBD oocytes (3 h) injected with *Ccnb* RNA or control *Gfp* RNA (*Cntrl)*. See Figure S4 for the over-expression of CCNB in oocytes. Representative images from at least three experiments of biologically different samples. (**b**) Quantification of protein expression, LMN A/C, and CDK1 phosphorylation. From three experiments of biologically different samples. Values obtained for the YF group were set as 100%. Data represent mean ± SD; ns non-significant; * *p* < 0.05; Student’s *t*-test. (**c**) Timing of NEBD of YF oocytes microinjected with *Gfp* (*n* = 46; black line) and *Ccnb* cRNA (*n* = 51; red line). From three experimental data sets of biologically different samples. Trend line is depicted by dot lines. Data represent mean ± SD. * *p* < 0.05, Student’s *t*-test. (**d**) Time from NEBD to PBE in oocytes from YF injected with RNA coding for *Gfp* (*n* = 46; t = 8:25 h) and *Ccnb* (*n* = 51; t = 7:52 h). Data represent mean ± SD and data are from at least three experiments of biologically different samples. Student’s *t*-test. (**e**) Representative Z-projections from the assessment of cold stable attachments of kinetochores (KT, CREST, green) to microtubule (MT, tubulin, red) of oocytes microinjected with control *Gfp* and *Ccnb* cRNA. Representative images are from three experiments of biologically different samples (scale bar, 10 µm). (**f**) The percentage of cold stable end-on MT to KT attachments in each group averaged over multiple cells (*n* ≥ 28) 6 h post-IBMX-wash. Kinetochore-MT end-on attachments were quantified. The morphology of kinetochores analyzed is specified in detail. Data represent mean ± SD. * *p* < 0.05, Student’s *t*-test.

It is known that the expression of CCNB is the limiting factor for the activation of MPF in oocytes prior to resumption of meiosis I [23,24]. It has been reported [25] that the slow increase in CDK1 activity during meiosis I acts as an intrinsic timing mechanism that ensures the appropriate stabilization of kinetochore attachments and thus guards the oocyte against chromosomal segregation errors. We examined whether the overexpression of CCNB affects the timing of meiotic progression. We overexpressed CCNB by microinjecting a cRNA encoding GFP-fused to CCNB into YF oocytes at the GV (0 h) stage (Figure S4). We also microinjected other GV oocytes with *Gfp* cRNA as a negative control. We found that, when experimenting with oocytes derived from the YF cohort, there was a significant increase (*p* < 0.05) in the levels of phosphorylated LMN A/C (Ser22) and phosphorylated CDK1 (Thr161) when microinjected with *Ccnb:gfp* cRNA, as measured 3 h post-IBMX-wash compared to the control group (Figure 6a,b). Live cell imaging of meiotic progression/maturation of such oocytes revealed that the increased expression of CCNB was also able to significantly accelerate overall meiotic progression, as evaluated by the timing of the NEBD and PBE (*p* < 0.05; Figure 6c,d). Specifically, oocytes injected with *Ccnb:gfp* extruded the polar body significantly earlier compared to the control group (Figure 6c). Thus, they mimicked the phenotype observed in the AF oocytes (Figure 1). Moreover, we also visualized cold-stable kinetochore-microtubule end-on attachments [25,26] and found that the oocytes overexpressing CCNB had a significantly higher rate of the kinetochore-microtubule end-on attachment than the controls at the 6 h post-IBMX-wash (*p* < 0.05; Figure 6e,f). These results suggest that artificially increasing MPF activity leads to a notably more rapid progression through meiosis I as exemplified by the production of stable kinetochore-microtubule attachments. 

Taken together, these experiments show we were able to mimic the faster meiotic progression observed in the AF oocytes, that itself stems from higher MPF activity. 

## 3. Discussion 

Here we have addressed the question of how meiosis I differs in oocytes from females of advanced reproductive age versus those originating from young females. The precise timing of the cell cycle is a prerequisite for the appropriate propagation of genomes. It is well accepted that, in human and mouse oocytes, the incidence of genomic instability and aneuploidy increases with maternal age [5,7,27,28,29,30]. Age-associated increase in aneuploidy [27,28,29,30] has been attributed, at least in part, to a faster progression through the first meiotic division in oocytes from aged females, which would affect the time available for proper chromosome congression prior to chromosome segregation [27]. The mouse is a useful model in which to study the effect of age on egg quality, including the molecular basis for observed age-associated increase in aneuploidy. 

We have found that oocytes from aged females resume meiosis and progress through meiosis I faster than the oocytes from young females. Our finding is consistent with findings of others [5,27,31,32,33] but opposes other findings reporting a lack of timing in oocytes from aged females [34,35]. In addition, we found that oocytes from aged females are significantly more meiotically competent than from young females, a further consistent observation [36]. The underlying reasons for the reported discrepancies related to the length of meiosis I in aged oocytes are not clear; however, they may have their origin in the methodologies employed to select meiotically competent GV oocytes and/or further differences in oocyte manipulation (e.g., the removal of cumulus cells and microinjection). Relating to our own data, we conclude that an increase in MPF activity during meiosis I temporary regulates acceleration of NEBD, the attachment of chromosomes, and cytokinesis events in aged oocytes. Moreover, our results are in agreement with previous findings [25] in which it is reported that premature increases of CDK1 kinase activity, induced by cyclin-B microinjection, are responsible for the precocious formation of stable kinetochore-microtubule attachments and lagging chromosomes during anaphase I, a condition that leads to aneuploidy. Thus, the increased presence of MPF components, as observed in our AF oocyte cohort, could clearly act in a similar manner to result in chromosome segregation errors during meiosis I. 

Surprisingly, we have also found that transcripts coding for MPF components are significantly overexpressed in the oocytes from aged females. However, it is important to recognize that the abundance of mRNA only represents one part of the regulation of gene expression and that selective translational regulation of transcripts can play a pivotal role. It has been previously reported that CCNB2 has a functional role during the prophase/metaphase transition of mouse oocyte maturation [37], and these data support our findings showing that the increased translational rate of CCNB2 transcripts in the oocytes from aged females might be associated with faster meiotic progression. Consistent with this prevailing view, we have also demonstrated elevated phosphorylation levels of CDK1 (Thr161), that in turn contributes to its MPF activity [38], in AF oocytes. Notwithstanding this observation, the upregulation of Cyclin B clearly plays a positive role in reinforcing CDK1/MPF activity and thus driving meiotic cycle progression. As such, our study correlates the resumption of meiosis with the synthesis of the regulatory subunit of MPF, namely cyclin B1/2, as supported by the fact that the level of MPF activity is known to depend on the amount of cyclin B present [22,39,40]. Thus, the fact that the MPF components in aged GV oocytes are apparently more expressed (but not necessarily fully active) and that they are then rapidly activated during their maturation (in AF versus YF oocytes) contributes to the observed acceleration of the AF oocytes meiotic progression. 

We show that both cyclins B are expressed and differentially occupy polyribosomes in the AF group. In addition to the increased expression of MPF components, which leads to accelerated meiotic progression, we also demonstrate that the key components of the translational machinery are more translated (associated with polysome fractions), which in turn is likely to positively affect general translation in AF oocytes. Indeed, our findings are in good agreement with published data describing increased numbers of ribosomes in oocytes from older females [41]. However, in connection to increased number of ribosomes, we have not observed increased rates of global translation. Nonetheless, our results suggest that there is increased translation of specific mRNAs related to specific translational machinery activity in AF oocytes, which may contribute to amplifying the roles of specific regulators [13,42]; mechanisms that could target specific mRNAs for translation and consequently affect meiotic progression rates. In addition to the increased number of ribosomes and translational regulators in oocytes from females of advanced age, it has also been reported [43,44] that the number of mitochondria is also increased in the oocytes and embryos derived from aged mouse and human subjects. 

We have also shown that the GV oocytes from female mice of advanced age have aberrantly formed nuclear envelopes, which strongly resemble the morphology of those in aged somatic cells [15,45]. In association with described precocious meiotic progression and increased MPF activity, we have shown that the nuclear lamina is also precociously dispersed in aged oocytes. We have previously reported that LMN A/C structures surround oocyte chromosomes post-NEBD, resembling an organelle-exclusion “spindle envelope” that acts as a diffusion barrier structure [46,47,48]. Such spindle envelopes are thought to confine spindle assembly and their mechanical disruption is reported to compromise precise and appropriate chromosome segregation in mitosis [47]. It is therefore possible that the lack of such a functioning spindle envelope in AF-derived oocytes contributes to increased aneuploidy rates observed. 

Taken together, our results can provide at least a partial explanation for the commonly recognized multifactorial phenomenon of age-related increase in oocyte aneuploidy on a molecular level. In addition, our study significantly contributes to the overall knowledge base concerning the molecular physiology of aged cells, including but not restricted to oocytes, and provides a solid foundation for further work related to the observed translational discrepancies between young and aged oocytes identified herein, and their functional interplay with meiotic progression/maturation. 

## 4. Material and Methods

### 4.1. Oocyte Cultivation, Treatment, and Microinjection

GV oocytes were obtained from CD1 mice 46 h after injection by 5 IU pregnant mare serum gonadotropin (PMSG, HOR 272, ProSpec, Rehovot, Israel). Oocytes were obtained from females in two distinct age categories: young females (YF) group (2 months old) and aged females (AF) group (12 months old). Oocytes were isolated in germinal vesicle stage (GV; 0 h) in transfer medium [49] supplemented with 100 µM 3-isobutyl-1-methylxanthine (IBMX, I5879, Sigma-Aldrich, Darmstadt, Germany) to prevent NEBD. Selected fully grown GV oocytes were denuded by pipetting and cultured in M16 medium (M7292, Sigma-Aldrich, Darmstadt, Germany) without IBMX at 37 °C, 5% CO_2_. Post-IBMX-wash (PIW) oocytes undergo nuclear envelope breakdown (NEBD) within 1 h; they reach metaphase I in 6 h and metaphase II in 12 h. For oocytes treatment, 20 µM roscovitin (186692-46-6, Cayman Chemical, Ann Arbor, MI, USA) was added 1 h PIW. GV oocytes were microinjected in the presence of the IBMX on inverted microscope Leica DMI 6000B (Leica Microsystems, Wetzlar, Germany) using TransferMan NK2 (Eppendorf, Hamburg, Germany) and FemtoJet (Eppendorf). Oocytes were injected with 20 ng/µL of in vitro transcribed (mMessage, Ambion, Thermo Fisher Scientific, Waltham, MA, USA) RNAs from plasmids (GFP, [50]; CCNB1, Dr. Martin Anger, Laboratory of Cell Division Control, IAPG, CAS) diluted in RNAse free water. Approximately 5 pL of RNA solution were injected into one oocyte. Microinjected oocytes were used for time-lapse microscopy, cold tubulin stability testing, and immunoblotting. All animal work was conducted according to Act No 246/1992 for the protection of animals against cruelty; from 25.09.2014 number CZ02389, issued by Ministry of agriculture.

### 4.2. Time-Lapse Microscopy

Oocytes were scanned with an inverted wide field microscope, Leica DMI 6000B (Leica Microsystems, Wetzlar, Germany), equipped with a chamber system (Pecon, Erbach, Germany), a Tempcontroller 2000-2 (Pecon), and a CO_2_ controller (Pecon). Cover-glass-based 4-well chambers (94.6190.402, Sarstedt, Nümbrecht, Germany) were used for live oocytes imaging. Oocytes were put into a 10 µL drop of M16 medium without IBMX and covered by approximately 1 mL of mineral oil (M8410, Sigma-Aldrich). The chamber was pre-tempered to 37 °C and 5% CO_2_. Images were captured every 5 min. Timing of the NEBD and polar body extrusion (PBE) were evaluated through time lapse movies.

### 4.3. Immunoblotting

Oocytes were washed in phosphate buffer saline (PBS, Sigma-Aldrich) with polyvinyl alcohol (PVA, Sigma-Aldrich) and frozen to −80 °C. An exact number of oocytes (15–30) were lysed in 10 μL of 1× Reducing SDS Loading Buffer (lithium dodecyl sulfate sample buffer NP 0007 and reduction buffer NP 0004, Thermo Fisher Scientific, Waltham, MA, USA) and heated at 100 °C for 5 min. Proteins were separated by gradient precast 4–12% SDS–PAGE gel (NP 0323, Thermo Fisher Scientific) and transferred to Immobilon P membrane (IPVD 00010, Millipore, Merck group, Darmstadt, Germany) using a semidry blotting system (Biometra GmbH, Analytik Jena, Jena, Germany) for 25 min at 5 mA cm^−2^. Membranes were blocked by 5% skimmed milk dissolved in 0.05% Tween-Tris buffer saline (TTBS), pH 7.4 for 1 h. After a brief washing in TTBS, membranes were incubated at 4 °C overnight with the primary antibodies diluted in 1% milk/TTBS (see Table S1). Secondary antibody Peroxidase Anti-Rabbit Donkey (711-035-152) or Peroxidase Anti-Mouse Donkey (715-035-151, Jackson ImmunoResearch, West Grove, PA, USA) was diluted 1:7500 in 1% milk/TTBS. The membranes were incubated in the secondary antibodies for 1 h at room temperature. Immunodetected proteins were visualized on films using ECL (Amersham, GE Healthcare Life Sciences, Barcelona, Spain). Films were scanned using a GS-800 calibrated densitometer (Bio-Rad Laboratories, CA, USA) and quantified using ImageJ (http://rsbweb.nih.gov/ij/). 

### 4.4. Measurement of Overall Protein Synthesis

To measure the overall protein synthesis, 50 μCi of ^35^S-methionine (Hartmann Analytics, Braunschweig, Germany) was added to methionine-free culture medium. Ten oocytes per sample were labeled for 2 h, then lysed in SDS-buffer, and loaded to SDS-polyacrylamide gel electrophoresis and transferred to an Immobilon P membrane using the semidry blotting system for 25 min at 5 mA cm^−2^ (the same materials as in Immunoblotting). The labeled proteins were visualized by autoradiography on FujiFilm (incubated at least 14 days in −80 °C), scanned using BAS-2500 Photo Scanner (FujiFilm Life Science, Tokyo, Japan) and quantified by ImageJ. GAPDH antibody was used as a loading control. 

### 4.5. Immunocytochemistry and Cold-Stable MT Assay

After cultivation, oocytes were fixed for 15 min in 4% paraformaldehyde (PFA, Alfa Aesar, Thermo Fisher Scientific, Waltham, MA, USA) in PBS/PVA. Oocytes were permeabilized in 0.1% Triton (X-100, Sigma-Aldrich) in PBS/PVA for 10 min, washed in PBS/PVA, and incubated overnight at 4 °C with primary antibodies (see Table S1). After washing in PBS/PVA, detection of the primary antibodies was performed by cultivation of the oocytes with relevant Highly Cross-Adsorbed Secondary Antibodies, Alexa Fluor 488, 594 or 647 conjugates (Thermo Fisher Scientific) diluted 1:250 for 1 h at room temperature. Oocytes were then washed two times for 15 min in PBS/PVA and mounted using a Vectashield Mounting Medium with DAPI (H-1200, Vector Laboratories, Burlingame, CA, USA). For the cold-stable MT assay, oocytes were matured for 6 h post-IBMX-wash and then were incubated for 15 min in 4 °C, fixed in 4% PFA/PVA, and stained for tubulin and CREST according to the immunocytochemistry protocol. Confocal images were collected as Z stacks at 0.3 µm intervals to visualize the entire meiotic spindle region. Samples were visualized using a Leica SP5 inverted confocal microscope (Leica Microsystems, Wetzlar, Germany). To classify kinetochore attachment status, images were scored around the same Z plane using the merged two-color confocal stack of CREST and MT images. Images were assembled in software LAS X (Leica Microsystems). 

### 4.6. Transmission Electron Microscopy 

Mouse oocytes in GV were washed three times in PBS/PVA and one time in 0.1 M Sorenson’s phosphate buffer (pH = 7.2) with PVA. Oocytes were fixed in 2.5% glutaraldehyde (G5882, Sigma-Aldrich) in 0.1 M Sorenson’s phosphate buffer for 1 h at room temperature. Fixed oocytes were transported to the Electron Microscopy facility at the Microscopy Centre of the Institute of Molecular Genetics, CAS. Fixative was removed and oocytes were centrifuged (5000× *g*/5 min) in 1% low-temperature melting agarose. Oocytes were embedded to Epon blocks and sliced by UltraCut6 (Leica Microsystems) to ultra-thin sections. Oocyte sections were imaged at 80 kV using FEI Morgagni 268 Transmission Electron Microscope with Olympus Megaview III Digital Camera EM (FEI Company, Hillsboro, OR, USA). 

### 4.7. RNA Isolation and RT-PCR 

RNA was extracted using a RNeasy Plus Micro kit (74034, Qiagen, Hilden, Germany) and genomic DNA was depleted using gDNA Eliminator columns. The quality and quantity of the isolated RNA was analyzed using the Bioanalyzer 2100 (Agilent, Santa Clara, CA, USA) system employing the RNA 6000 Pico kit (5067-1513, Agilent). RT-PCR was then carried out using the Rotor-Gene 3000 (Biocompare, South San Francisco, CA, USA) and the OneStep RT-PCR Kit (210210, Qiagen) and SybrGreen I (S7563, Thermo Fisher Scientific) according to manufacturers’ provided protocols. Gene/transcript specific RT-PCR primers were designed with an annealing temperature of 58 °C (see Table S2). The reaction condition for reverse transcription was as follows: 50 °C/30 min, then initial activation at 95 °C/15 min, followed by 40 PCR amplification cycles (95 °C/15 s, 58 °C/20 s, 72 °C/30 s) and 72 °C/10 min. Quantification analyses were performed using a dynamic amplification efficiency determination for each amplification run as provided in the comparative quantification function with the Rotor-Gene RG-3000 software. The exact amplification efficiencies were assessed in each tube, and a mathematic model was applied for the derived calculation of the relative gene expression in the control (YF).

### 4.8. Polysome Fractionation and RNA Extraction

Prior to oocyte collection, 100 µg/mL of cycloheximide (CHX, 01810, Sigma-Aldrich) was added for 10 min. Next, 200 oocytes (per sample) were washed three times in PBS/PVA supplemented with CHX and frozen at –80 °C in low-binding tube (Eppendorf). To disrupt the zona pellucida and lysate the oocytes, 250 µL of zirconia-silica beads (11079110z, BioSpec, Bartlesville, OK, USA) were added to the tube with frozen oocytes together with 350 µL of lysis buffer (10 mM Hepes, pH 7.5; 62.5 mM KCl; 5 mM MgCl_2_; 2 mM DTT; 1% TritonX-100; 100 µg/mL of CHX supplemented with Complete-EDTA-free Protease Inhibitor (05 056 489 001 3, Roche Diagnostics GmbH, Mannheim, Germany) and Ribolock 20 U/mL (EO0381, Thermo Fisher Scientific)). Oocytes were disrupted in the mixer mill apparatus MM301 (shake frequency 30, total time 45 s, Retsch, Haan, Germany). Lysates were clarified by centrifugation at 8000× *g* for 5 min at 4 °C). Supernatants were loaded onto 10–50% linear sucrose gradients containing 10 mM Hepes, pH 7.5; 100 mM KCl; 5 mM MgCl_2_; 2 mM DTT; 100 μg/mL CHX; Complete EDTA-free (1 tablet/100 mL); and 5 U/mL Ribolock. Centrifugation was carried out using Optima L-90 ultracentrifuge (Beckman Coulter, Brea, CA, USA) at 35,000× *g* for 65 min at 4 °C. Polysome profiles were recorded using ISCO UA-5 UV absorbance reader. We monitored the overall quality of the polysome fractionation experiment by an inclusion of a parallel HEK293 cells sample. Ten equal fractions were recovered from each sample and subjected to RNA isolation by Trizol reagent (Sigma-Aldrich). Each fraction was then tested by qPCR with 18S and 28S rRNA-specific primers in LightCycler480 (Roche) to reconstruct a distribution of non-polysomal and polysomal RNA complexes in each oocyte—specific profile.

### 4.9. Library Preparation, RNA Sequencing and Data Analysis

Fractions corresponding to polysomal and non-polysomal part, respectively, were pulled together. These sub-samples were concentrated to 16 µL of Clean & Concentrator-5 (R1014, Zymo Research, Irvine, CA, USA) and ribosomal RNA was removed from them by Ribozero-Gold (MRZG12324, Illumina, San Diego, CA, USA). Afterwards, RNA was turned into cDNA and amplified by using the REPLI-g WTA Single Cell Kit (150063, Qiagen). Finally, cDNA was tagmented and libraries were prepared using the Nextera DNA Library Prep Kit (FC-121-1030, Illumina). Sequencing was performed in Centro Nacional de Analisys Genomico facility (CNAG, Barcelona, Spain). Samples were sequenced by HiSeq 2500 (Illumina) as 150 bp paired-end. Reads were trimmed using Trim Galore! v0.4.1 and mapped to the mouse GRCm38 genome assembly using Hisat2 v2.0.5. Gene expression was quantified as fragments per kilobase per million (FPKM) values in Seqmonk v1.40.0. Functional annotation was performed using GOrilla [51,52] with ranked lists of genes detected in polysomal fractions (FPKM > 0.1).

### 4.10. Kinase Assay 

Kinase activities of CDK1 (H1) and MAPK (MBP) were determined in a single assay via their capacity to phosphorylate external substrates histone H1 and myelin basic protein (MBP), respectively [53]. Fifteen oocytes per sample were collected and lysed in 5 µL of lysis buffer (10 µg/mL leupeptin, 10 µg/mL aprotinin, 10 mM p-nitrophenyl phosphate, 20 mM β-glycerophosphate, 0.1 mM Na_3_VO_4_, 5 mM EGTA, 1 mM benzamidine, 1 mM AEBSF) by three cycles of freezing/thawing. Next, 5 µL of double kinase buffer (60 µg/mL leupeptin, 60 µg/mL aprotinin, 24 mM p-nitrophenyl phosphate, 90 mM β-glycerophosphate, 4.6 mM Na_3_VO_4_, 24 mM EGTA, 2 mM benzamidine, 2 mM AEBSF, 24 mM MgCl_2_, 0.2 mM EDTA, 4 mM NaF, 1.6 mM DTT, 0.2% (*w*/*v*) polyvinyl alcohol, 40 mM MOPS pH 7.2, 2.2 µM protein kinase inhibitor (P0300, Sigma-Aldrich), 1 mg/mL MBP (M1891, Sigma-Aldrich), 0.5 mg/mL histone H1 (10223549001, Roche), 0.6 mM ATP, 1 mCi/mL [γ-^32^P] ATP (Hartmann Analytic, Braunschweig, Germany) was incubated with the lysed sample for 30 min at 30 °C. The reaction was terminated by addition of 12.5 μL of double-strength concentrated reducing sample buffer [54]. The phosphorylated substrates were resolved on 15% SDS-PAGE gel, the gel was stained with Coomassie Brilliant Blue R250 (27816, Sigma-Aldrich), dried and exposed to an intensifying screen in the exposure cassette for 20 h. Phosphorylated substrates were visualized using a FujiFilm BAS-2500 Photo Scanner and the kinase activity was quantified using Aida Image Analyzer software (Elysia Raytest, Angleur, Belgium). 

### 4.11. Statistical Analysis 

Mean and standard deviation (±SD) values were calculated using MS Excel. Statistical significance of the differences between the groups was tested using Student’s *t*-test (PrismaGraph5) and *p* <0.05 was considered as statistically significant (marked by asterisks: * *p* < 0.05; ** *p* < 0.01; *** *p* < 0.001). 

## Supplementary Materials

Supplementary materials can be found at http://www.mdpi.com/1422-0067/19/9/2841/s1. Figure S1. Localization of LMN A/C during oocyte maturation. (**a**) Representative confocal images from immunocytochemistry (ICC) showed localization of LMN A/C (red) and phosphorylated LMN A/C Ser22 (green) during oocyte maturation (GV 0 h; NEBD 3 h; MI 6 h, MII 12 h). Cortex of oocytes is depicted by white dashed line. DNA, blue and scale bar, 10 µm. (**b**) Co-localization of LMN A/C (Ser22) (green) and the spindle (tubulin, red). DNA, blue and scale bar 10 µm. (**c**) Localization of LMN A/C (red) during oocyte meiotic maturation. DNA, blue and scale bar 10 µm. Arrowhead marks polar body. Figure S2. Transmission electron microscopy of oocyte nuclei from females of different age. (**a**) Representative images of the nucleus from YF and AF oocytes. The images in the right panels show nuclear membrane highlighted with red line. Scale bar 10 µm. (**b**) Measurement of nuclear membrane circumference of oocytes from the YF and the AF group. From two experiments of biologically different samples (n ≥ 8). Data represent mean ± SD. ** *p* < 0.01, Student’s *t*-test. (**c**) Detail of nuclear lamina from AF and YF oocytes. Representative images are from two experiments from biologically different samples (bar, 1 µm). The images in the right panels show nuclear membrane highlighted with red line. Figure S3. Total RNA amount and global translational activity is not different between YF and AF groups. (**a**) Quantification of total RNA by Agilent 2100 Bioanalyzer in the oocytes from different age groups. From 10 experiments of biologically different samples. Data represent mean ± SD. ns, non-significant, Student’s *t*-test. (**b**) ^35^S-Methionine incorporation during meiotic progression of oocytes from YF and AF groups. Representative images are from three experiments of biologically different samples. (**c**) Quantification of ^35^S-Methionine incorporation in the oocytes from different groups. From three experiments of biologically different samples. Values obtained for the YF group were set as 100%. Data represent mean ± SD, *ns*, non-significant, Student’s *t*-test. Figure S4. Induced expression of the CCNB in the oocytes. Oocytes injected with control *Gfp* (*Cntrl*) and *Ccnb* RNA. See Figure 6a for the effect of the overexpression. WB analysis of samples using CCNB antibody. Arrowhead depicts endogenous CCNB and arrow GFP tagged CCNB protein. GAPDH was used as a loading control. From three experiments of biologically different samples. Table S1. Primary antibodies used for WB and ICC in the study. Table S2. Primers used in the study for RT-PCR.
