# Supplementary material for: Increased Expression of Maturation Promoting Factor Components Speeds Up Meiosis in Oocytes from Aged Females
Source: Int J Mol Sci. 2018 Sep 19;19(9):2841. doi: 10.3390/ijms19092841 (PMC6164426; doi:10.3390/ijms19092841)
Supplement: Supplementary file 1 [file ijms-19-02841-s001.pdf]

Supplementary Material:

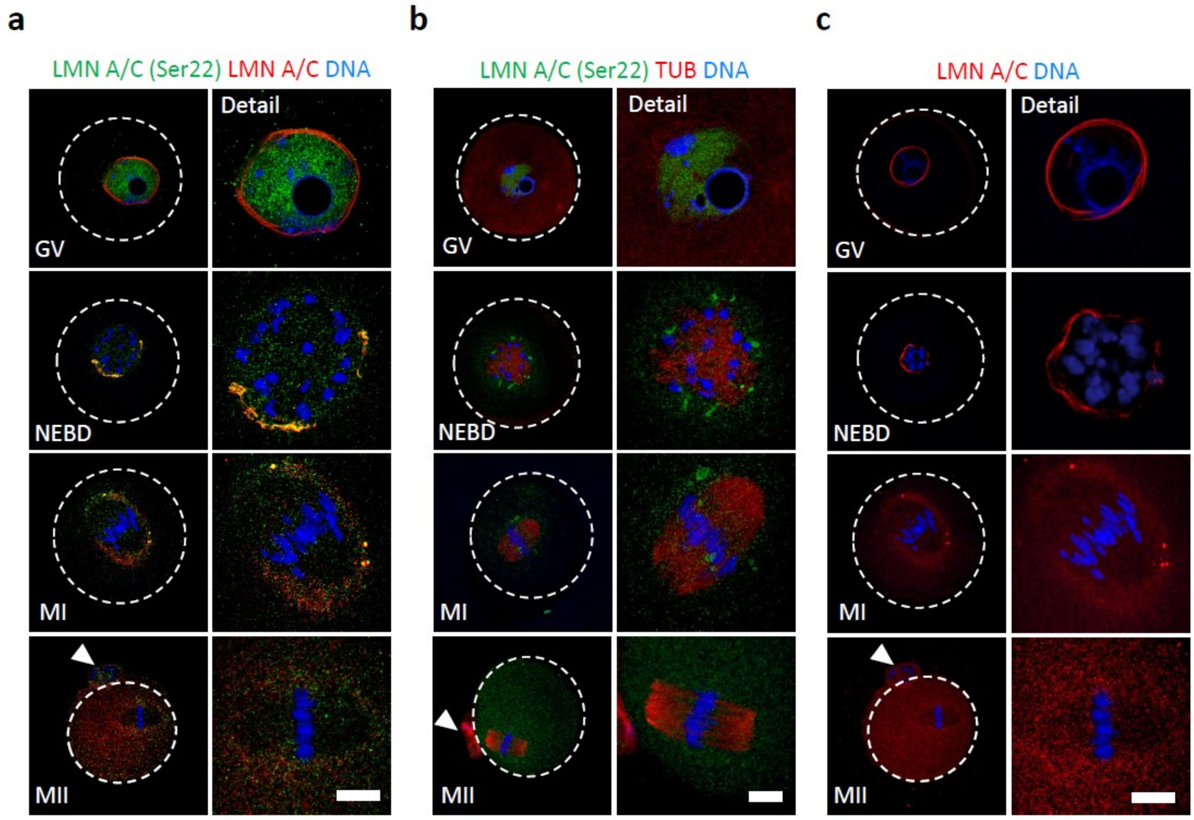

Figure S1. Localization of LMN A/C during oocyte maturation. (**a**) Representative confocal images from immunocytochemistry (ICC) showed localization of LMN A/C (red) and phosphorylated LMN A/C Ser22 (green) during oocyte maturation (GV 0 h; NEBD 3 h; MI 6 h, MII 12 h). Cortex of oocytes is depicted by white dashed line. DNA, blue and scale bar, 10 µm. (**b**) Co-localization of LMN A/C (Ser22) (green) and the spindle (tubulin, red). DNA, blue and scale bar 10 µm. (**c**) Localization of LMN A/C (red) during oocyte meiotic maturation. DNA, blue and scale bar 10 µm. Arrowhead marks polar body.

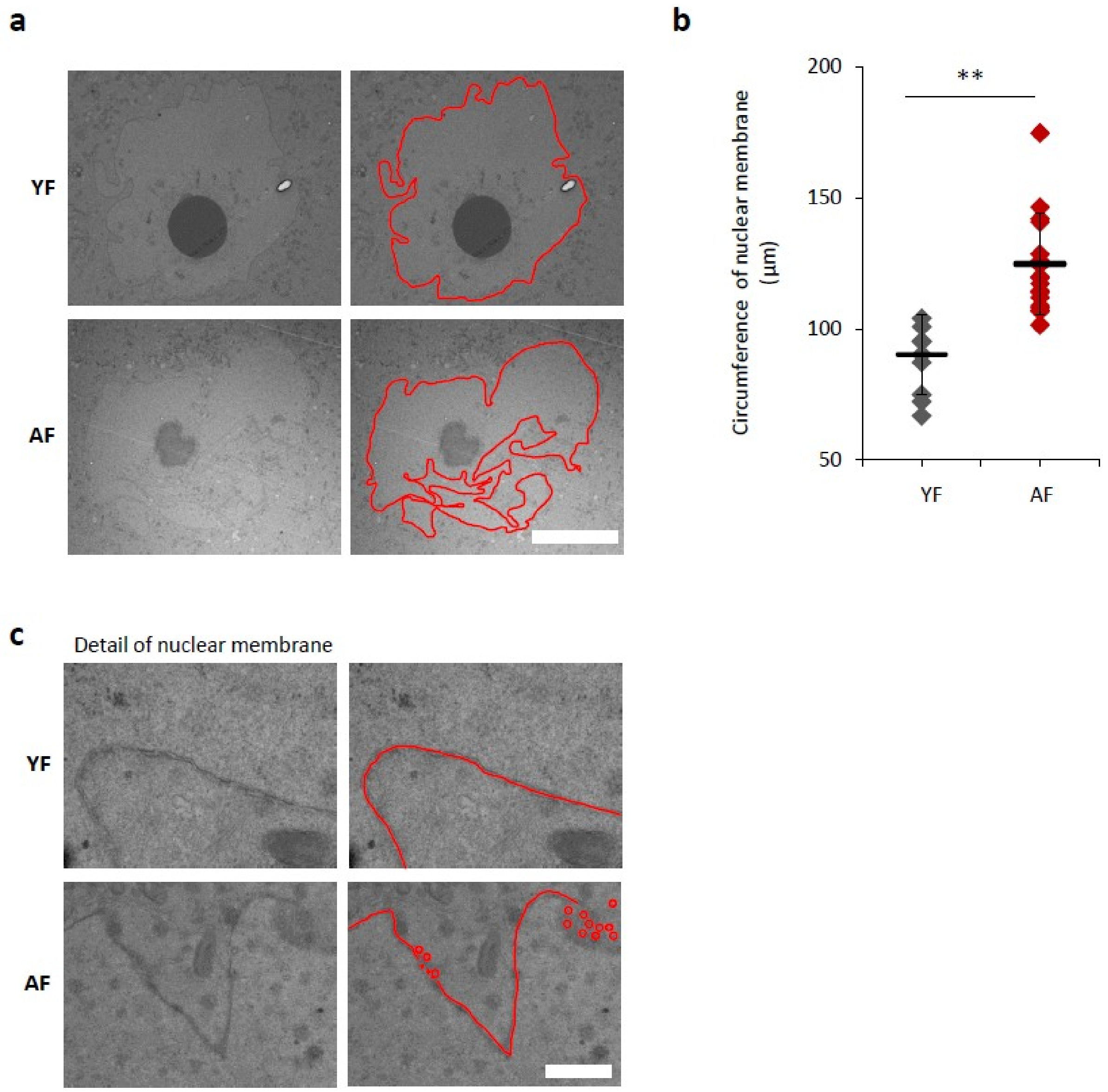

Figure S2. Transmission electron microscopy of oocyte nuclei from females of different age. (**a**) Representative images of the nucleus from YF and AF oocytes. The images in the right panels show nuclear membrane highlighted with red line. Scale bar 10 µm. (**b**) Measurement of nuclear membrane circumference of oocytes from the YF and the AF group. From two experiments of biologically different samples ($n \geq 8$). Data represent mean ± SD. ** $p < 0.01$, Student's *t*-test. (**c**) Detail of nuclear lamina from AF and YF oocytes. Representative images are from two experiments from biologically different samples (bar, 1 µm). The images in the right panels show nuclear membrane highlighted with red line.

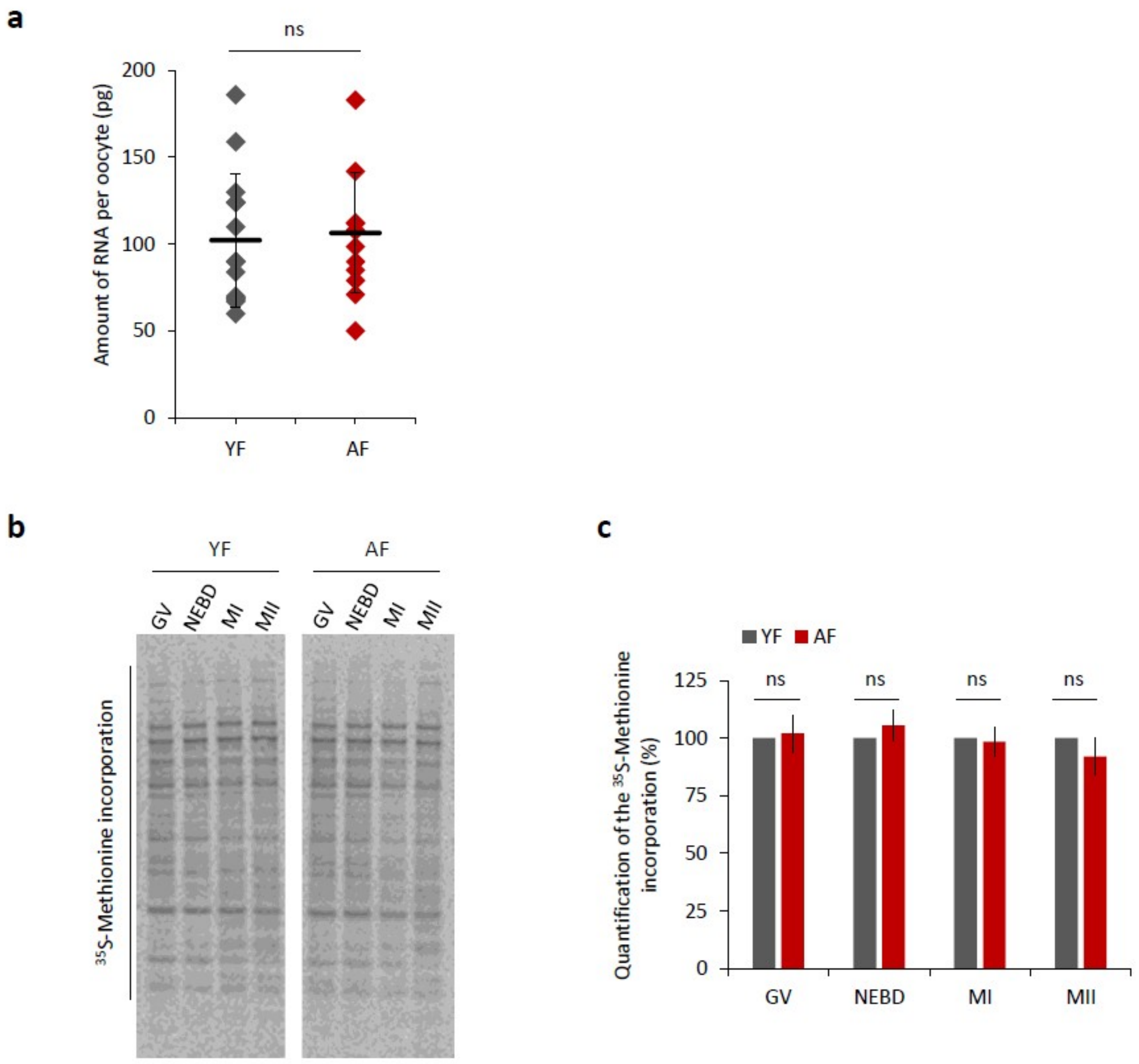

Figure S3. Total RNA amount and global translational activity is not different between YF and AF groups. (**a**) Quantification of total RNA by Agilent 2100 Bioanalyzer in the oocytes from different age groups. From 10 experiments of biologically different samples. Data represent mean ± SD. ns, non-significant, Student's *t*-test. (**b**) ^{35}S-Methionine incorporation during meiotic progression of oocytes from YF and AF groups. Representative images are from three experiments of biologically different samples. (**c**) Quantification of ^{35}S-Methionine incorporation in the oocytes from different groups. From three experiments of biologically different samples. Values obtained for the YF group were set as 100%. Data represent mean ± SD, *ns*, non-significant, Student's *t*-test.

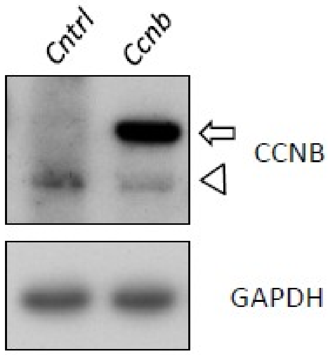

Figure S4. Induced expression of the CCNB in the oocytes. Oocytes injected with control *Gfp* (*Cntrl*) and *Ccnb* RNA. See Figure 6a for the effect of the overexpression. WB analysis of samples using CCNB antibody. Arrowhead depicts endogenous CCNB and arrow GFP tagged CCNB protein. GAPDH was used as a loading control. From three experiments of biologically different samples.

Table S1. Primary antibodies used for WB and ICC in the study.

Primary antibodies	Cat. No., company	Western Blot (WB)	Immunocytochemistry (ICC)
Acetylated Tubulin	T6793, Sigma-Aldrich	not used	1:150
CDK1	MA5-11472, Thermo Fisher Scientific	1:500, 1% milk	not used
CDK1 (Thr161)	9114, Cell Signalling Technology	1:500, 1% milk	not used
CREST	HCT-0100, ImmunoVision	not used	1:1000
Cyclin B	MS-338-PO, Thermo Fisher Scientific	1:500, 1% milk	not used
GAPDH	G9545, Sigma-Aldrich	1:30000, 1% milk	not used
Lamin A/C	SAB4200236, Sigma-Aldrich	1:2000, 1% milk	1:150
Lamin A/C (Ser 22)	2026, Cell Signalling Technology	1:500, 1% milk	1:150

Table S2. Primers used for RT-PCR.

Official symbol (gene)	Forward 5´- 3´	Reverse 5´- 3´	Gene Bank ID	Product size (bp)	Annealing temperature °C
18S	CTCAACACGGGAAACCTCAC	CGCTCCACCAACTAAGAACG	NR_003278.3	110	58
28S	CTAAATACCGGCACGAGACC	TTCACGCCCTCTTGAACTCT	NR_003279.1	88	58
Ccnb1	ACAGCTGGTCGGTGTAACG	TGCACCATGTCGTAGTCCAG	NM_172301.3	282	58
Ccnb2	CCGACGGTGTCCAGTGATTT	AGGTTTCTTCGCCACCTGAG	NM_007630.2	141	58
Cdk1	GAACGGCTTGGATTTGCTCTC	AGCAGACAGGGACATCCATC	NM_007659.3	108	58
Gapdh	CGGGAAGCCCATCACGATTT	GGTCATGAGCCCTTCCACAA	XM_001476707.5	280	58
